# Correction of Rotational Eccentricity Based on Model and Microvision in the Wire-Traction Micromanipulation System

**DOI:** 10.3390/mi14050963

**Published:** 2023-04-28

**Authors:** Yuezong Wang, Daoduo Qu, Shengyi Wang, Jiqiang Chen, Lina Qiu

**Affiliations:** Faculty of Materials and Manufacturing, Beijing University of Technology, Beijing 100124, China; yaozongw@bjut.edu.cn (Y.W.); shengyihappy@emails.bjut.edu.cn (S.W.); chenjiqiang2022@emails.bjut.edu.cn (J.C.); qiulina2020@emails.bjut.edu.cn (L.Q.)

**Keywords:** coreless coil, eccentricity model, microvision, measurement, correction, micromanipulation

## Abstract

In the realm of automatic wire-traction micromanipulation systems, the alignment of the central axis of the coil with the rotation axis of the rotary stage can be a challenge, which leads to the occurrence of eccentricity during rotation. The wire-traction is conducted at a micron-level of manipulation precision on micron electrode wires; eccentricity has a significant impact on the control accuracy of the system. To resolve the problem, a method for measuring and correcting the coil eccentricity is proposed in this paper. First, models of radial and tilt eccentricity are established respectively based on the eccentricity sources. Then, measuring eccentricity is proposed by an eccentricity model and microscopic vision; the model is used to predict eccentricity, and visual image processing algorithms are used to calibrate model parameters. In addition, a correction based on the compensation model and hardware is designed to compensate for the eccentricity. The experimental results demonstrate the accuracy of the models in predicting eccentricity and the effectiveness of correction. The results show that the models have an accurate prediction for eccentricity that relies on the evaluation of the root mean square error (RMSE); the maximal residual error after correction was within 6 μm, and the compensation was approximately 99.6%. The proposed method, which combines the eccentricity model and microvision for measuring and correcting eccentricity, offers improved wire-traction micromanipulation accuracy, enhanced efficiency, and an integrated system. It has more suitable and wider applications in the field of micromanipulation and microassembly.

## 1. Introduction

Micromanipulation is the operation of objects with a size range from micron to submillimeter. It is usually composed of a motion control system and a micromanipulator, and its operating accuracy can typically reach micron or even submicron or nanometer levels [1]. Thus, this kind of system has been popularly applied in microassembly [2], microinjection [3], bioengineering [4], minimally invasive surgery [5], and other microscopic fields.

A micro coreless motor is a typical sample of micromanipulation, and it is also an emerging product produced by the coreless coil. It has the outstanding characteristics of being energy-saving, offering sensitive control, having high precision, being lightweight, etc., and they are widely used in aerospace, medical devices, intelligent robots and other high-tech fields [6,7]. In its manufacturing process, the wire-traction technology of the coreless coil is an important link. Prior to that, the wire-traction was primarily manually adjusted under a microscope. Because the diameter of the electrode wire was only several tens of microns, the wire was soft and its bearing capacity was tiny, which led to the difficulty of manual operation. Therefore, the efficiency, quality, and consistency were defective to a certain extent.

In view of this problem, Wang et al. [8,9,10] designed an automatic micromanipulation system for coil wire-traction based on microvision. In the system, the electrode wire was clamped by the microgripper and guided by microvision to make the wire lie on the pad in order to meet the requirements of automatic traction. Specifically, the coil was fixed on the cylindrical stepped base to facilitate continuous pick-up operation, and the base was fixed at the upper surface center of the rotary by a mechanical limit for multiangle manipulation. Due to systematic errors such as machining and assembly, it is difficult to ensure that the coil center coincides with the rotary center. Therefore, eccentricity will occur when the coil rotates. It is found that the coil’s deviation from the initial position can reach tens to hundreds of microns during the rotation. The micromanipulation system operates on micron-level objects. The dimension of the electrode wire and the tip of the microgripper are small, and the whole operation is also limited in a narrow area. The eccentricity decreases the operating accuracy of the system and leads to wire-traction failure. The method of wire-traction micromanipulation and the structure of the microgripper are widely concerned. However, improving control accuracy in the micromanipulation system is more significant, which has rarely been investigated. Therefore, it is particular important to measure and correct eccentricity in the wire-traction system.

In the study of eccentricity caused by machining and assembly errors, the installation eccentricity of the circular grating used for the angle sensor is taken as a representative example. A variety of works have been proposed to measure and correct its eccentricity bias. Geckeler et al. [11] proposed a self-calibration method based on a Fourier-based algorithm through a suitable geometric arrangement of multiple reading heads. The angle measurement error due to lateral shifts, graduation error, the reading heads’ positions, and nonuniformity were analyzed. Li et al. [12] analyzed the angular positioning error of a rotary stage in a machine tool and open-loop controlled system by causing the tilt and radial motions of the rotation axis of a circular grating. In addition, the multiple reading heads with double-contact-type dial indicators were used to measure and eliminate the geometric error of the rotary stage to enhance the angle measurement accuracy. Yu et al. [13] proposed a compensation method based on a single reading head to calibrate the eccentricity error of circular grating instead of multi reading heads. The mathematic models of errors were developed, and a compensation function was constructed. In addition, the proposed method achieved the same accuracy level as the hardware method. Du et al. [14] proposed an eccentricity error model and inclination error model based on the angle measurement error of the circular grating. The calibration and compensation methods based on the proposed error model could effectively compensate its angle measurement error with a single reading head and obtain a high-precision measurement angle. Jia et al. [15] analyzed the angle measurement error caused by the installation eccentricity of a grating disk and the radial error of a rotating shaft, and proposed to detect the error through a vision system and image processing technology.

The measurement and correction for eccentricity error was also applied to the rotating spindle of some equipment. Chen et al. [16] proposed using a laser displacement sensor to measure the eccentricity error of the spindle and using a piezoelectric driver to apply micro displacement on the grinding wheel to realize the alignment with the spindle. Lou et al. [17] proposed a model for predicting the angular positioning error of a rotational mandrel caused by the coaxiality error of the mandrel and the radial error motion of the spindle. The coaxiality and radial error were measured and calibrated by the use of an optical polygon and an autocollimator. Lou et al. [18,19] proposed an embedded sensor system for the real-time measurement of two radial and three angular error motions of a rotary stage. The geometrical relationship between the four scanning heads and radial error motions was proposed and verified. The rotational angle and radial error motions of the shaft were measured using an encoder with multiple scanning heads. Huang et al. [20] used multi laser displacement sensors to measure the eccentricity error between a grinding wheel and a spindle through an inversion method. Finally, the eccentricity was adjusted by three piezoelectric actuators installed inside a flange.

In summary, the existing research status to measure and correct the eccentricity error are summarized as follows: (1) The analysis of the eccentric source is not comprehensive enough, the eccentricity model is not taken into account, or the model is not in close association with the measurement and correction. (2) The measurement technique of eccentricity mostly relies on sensors, which are contactable, such as a probe displacement indicator, and noncontactable, such as a reading head, laser displacement sensor, and photoelectric autocollimator, etc. Probe-type and laser displacement sensors are easily affected by the shape error accuracy of the measured object surface. The combination of a photoelectric autocollimator and reflective prism has high measurement accuracy, but its cost and installation requirements are high, and it is applicable to large measured objects. The use of multiple sensors for measurement enhances the accuracy compared to a single sensor; however, it may lead to additional error sources and increased system complexity. (3) Vision-based technology presents several advantages, such as high accuracy, time-saving features, and low cost. It is also expected to become the future trend for detecting eccentricity bias for multiscale objects due to its ease of being embedded in various systems.

In this work, a method of eccentricity measurement and correction based on the eccentricity model and microvision detection is proposed. Firstly, the main sources of coil eccentricity are analyzed, and they are verified by the establishment of corresponding eccentric models. A microscopic vision system and image processing algorithm are used to collect and process microscope images of the coil in order to calibrate model parameters. Then, the eccentricity value can be predicted by the eccentricity models. A coarse–precise method combining the compensation model and hardware is designed to correct the eccentricity. Finally, the prediction accuracy of the eccentricity models and the effectiveness of the correction method are verified by experiments. The results show that the proposed method can effectively solve the eccentric problem in a wire-traction system, as well as enhance the manipulation accuracy of the system. The contribution of this paper is mainly reflected in three aspects:

(1) The eccentricity model and microvision for measuring eccentricity have high measurement accuracy and speed;
(2) The coarse–precise correction based on model and hardware has better compensation performance;
(3) The approach may be more suitable for micromanipulation, and other highly integrated systems, due to its embeddability.

The remainder of this article is organized as follows: in Section 2, the materials and methods are designed; and, in Section 3, the experimental results and discussion are carried out. In Section 4, the conclusions are derived.

## 2. Materials and Methods

In this section, the proposed method for the measurement and correction of coil eccentricity is designed. The automatic wire-traction micromanipulation system and its traction method are introduced, and the sources of rotational eccentricity are analyzed from the view of engineering in Section 2.1. The eccentricity value changes periodically with the rotation of the rotary shift. Therefore, the establishment of eccentricity models can reveal its laws and provide ideas for measurement and correction, which are discussed in Section 2.2. In Section 2.3, an image processing algorithm is used to detect and calibrate model parameters to predict the eccentricity. In addition, it also has a guiding significance for the eccentricity correction in Section 2.4.

### 2.1. The Wire-Traction System and Its Rotational Eccentricity

The previously mentioned automatic micromanipulation system was designed for the wire-traction of the micro coreless coil. The wire-traction system and the coil are shown in Figure 1. The coil is the core component of the coreless micro motor. It is composed of a barrel winding coil, rotating shaft, pad, and electrode wire. The outer diameter of the coil *Ф1* and its height *H* are about 2–3 mm and 12 mm, respectively; the outer diameter of electrode wire *Ф2* and its length *L* are about 0.05–0.07 mm and 1.5–2 mm, respectively; and the outer diameter of coil center column *Ф3* is about 1 mm. The top of the coil usually contains three pieces of electrode wires and pads. The coreless coil needs to undergo a series of process treatments before it can be assembled into the motor to form a product. It is particularly important to pull the electrode wire to the corresponding pad (called wire-traction). Thus, the microgripper designed for the wire-traction is shown in Figure 2. Its overall dimensions are the following: the length *L* and width *W* are designed to be 15.3 mm and 15.0 mm, respectively. Its tip envelope area is designed to be elliptical, and its dimensions are the following: the long axis *l*_1_ and the short axis *l*_2_ are 0.20 mm and 0.14 mm, respectively. The microgripper adopts the processing technology of high-precision laser cutting, its material is 304 stainless steel, and the overall thickness is 0.20 mm. In addition, the system also includes a microvision system composed of two complementary metal oxide semiconductor (CMOS) cameras to detect electrode wires, as well as a five-axis motion control system. The sensors are used to measure eccentricity error as part of the final comparative experiment.

According to the system mentioned above, the designed automatic wire-traction procedure is shown in Figure 3a–c, which can be summarized as follows:

Step 1: Wire detecting and returning. The visual system detects the coordinate position of wires (A, B, C). The target wire to be operated is returned to the ROI (region of interest) by the driven rotary, which represents a successful detection. The size of ROI is related to the clamping area.

Step 2: Capture and clamping of electrode wires. The coil is driven to move a regular distance to send the electrode wire to the clamping area of the microgripper in order to complete the capturing and clamping of the wire.

Step 3: Traction and release of electrode wires. The coil is driven to rotate, and the wire is pulled by using the contact reaction force, which is then released to the corresponding pad. Repeating above steps, the wire-traction can be completed.

In fact, the coil will generate eccentricity during rotation. Its actual eccentricity is shown in Figure 4a. The offset of the coil center line is relative to the ideal rotation axis, which generates a radial error. It also generates tilt error due to its tilt angle, which is caused by poor flatness of the base. They are the main eccentricity error sources in this system, and the coil’s eccentric trajectory is shown in Figure 4b. It is found that the error of the coil deviating from the initial position during rotation can reach tens or even hundreds of microns. This will cause the wire to fluctuate in the plane. Thus, it will affect the normal process of wire-traction, as shown in Figure 3d,e. Due to the existence of eccentricity, it will cause the electrode wire to deviate from the ROI for detection and result in a failure detection. The clamping area is tiny, and the change in the *L* easily causes the microgripper to fail to capture the wire; in addition, the wire cannot be accurately pulled to the center of the pad, or else the tip of the microgripper starts to collide and fracture because of closing to the shaft of the coil. In conclusion, the coil eccentricity detrimentally impacts the control accuracy of the system, thus compromising the reliability and stability of the whole system. Consequently, it is of paramount importance to measure and correct the eccentricity of the coreless coil in the wire-traction system.

### 2.2. Eccentricity Measurement by Model

#### 2.2.1. Radial Eccentricity Model

Without considering the tilt angle, the radial eccentricity caused by the coil center offset can be established, as shown in Figure 5a. For the convenience of analysis, the following coordinate system was established. Under the pixel coordinate system *UV*, the actual rotation center of the rotary stage is set as the origin *O*, the straight line from the origin *O* to the zero-point *A* of the rotary stage is set as the *X*-axis, and the line passing through the origin *O* and perpendicular to the *X*-axis is set as the *Y*-axis. The *XOY* coordinate system is established. The geometric projection center *o* of the coil is usually not coincident with the rotation center *O* in the *UV*. The distance between these two points is the coil eccentricity *e*, where the outline of green dotted line represents the base circle of the rotary stage. The outline of the blue dotted line indicates the outer circle of the coil. The vector from the actual rotation center *O* to the coil geometric center *o* is defined as the eccentricity vector, the magnitude of which is the eccentricity *e*, and the direction is the initial eccentricity angle *α*. The red track circle formed by the end of the eccentric vector rotating around the rotation center is the eccentricity circle in the radial eccentricity of the coil.

When the rotary stage drives the coil to rotate, the coil center moves to the next position *o*′. *θ* is the actual rotation angle of the rotary stage, and ***δ_e_*** is the eccentric vector at this position relative to the initial position. Obviously, ***δ_e_*** = ***Oo*′** − ***Oo***; assuming that the initial coordinate of the coil geometric center *o* is (*x*_0_,*y*_0_), and the coordinate of the actual rotation center *O* of the coil is (*u*,*v*), then we obtain the following:

The initial eccentricity angle of the coil is:

(1) $$\alpha =\arctan\frac{y_{0}-v}{x_{0}-u}$$

The eccentric circle equation for one rotation of the coil is:

(2) $${\left(x-u\right)}^{2}+{\left(y-v\right)}^{2}=e^{2}={\left(x_{0}-u\right)}^{2}+{\left(y_{0}-v\right)}^{2}$$

The components of radial eccentricity *δ_e_* relative to initial position on *X*-direction and *Y*-direction are expressed as:

(3) $$\left\{\begin{array}{l} \delta_{eX}=e\left[\cos(\alpha +\theta )-\cos(\alpha )\right]\\ \delta_{eY}=e\left[\sin(\alpha +\theta )-\sin(\alpha )\right] \end{array}\right.$$

Equation (3) shows that the radial eccentricity is not only related to the eccentricity value *e* and the initial eccentricity angle *α*. Therefore, the error can be calculated by the rotation center and initial position. This provides ideas for the later analysis of the tilt and comprehensive eccentricity. However, since the tilt angle always exists, the radial eccentricity without considering tilt eccentricity cannot be calculated directly.

#### 2.2.2. Tilt Eccentricity Model

Without considering the radial eccentricity, the tilt model caused by the coil tilt was established, as shown in Figure 5b. The spatial coordinate system of the rotating stage is *OXYZ*, the spatial coordinate system of the coil is *oxyz*, the *z*-axis of the coil center line passes through the origin *O* of the rotating coordinate system, and the angle *β* between the straight-line *Oo* and the rotation *Z*-axis is the tilt angle of the coil. The distance between the geometric center *o* and the origin *O* of the rotating coordinate system is *l*, which is approximately equal to the height of the coil. The projection of the inclined coil on *XOY* is a blue dotted line ellipse with the center *o*′. The analysis of the coil’s tilt error in *UV* is similar to Section 2.2.1. Assuming that the initial coordinate of the coil geometric center *o* is (*x*_0_,*y*_0_), and the coordinate of the actual rotation center *O* is (*u*,*v*), then we obtain the following:

The initial eccentricity angle of coil:

(4) $$\alpha^{\prime }=\arctan\frac{y_{0}-v}{x_{0}-u}$$

The eccentric circle equation for one rotation of the coil is:

(5) $${\left(x-u\right)}^{2}+{\left(y-v\right)}^{2}=l^{2}\sin^{2}\beta$$

The components of tilt eccentricity *δ_β_* relative to initial position after rotation on *X*-direction and *Y*-direction are:

(6) $$\left\{\begin{array}{l} \delta_{\beta X}=l\sin\beta \left[\cos(\alpha^{\prime }+\theta )-\cos(\alpha^{\prime })\right]\\ \delta_{\beta Y}=l\sin\beta \left[\sin(\alpha^{\prime }+\theta )-\sin(\alpha^{\prime })\right] \end{array}\right.$$

Equation (6) shows that the tilt eccentricity *δ_β_* is related to the coil tilt angle *β* and the initial eccentricity angle *α*′. The eccentricity angle can be calculated from the coordinate of the initial center of the coil. Compared with the radial eccentricity, the tilt eccentricity can be calculated directly after determining the tilt angle *β*, rotation center, and coil initial position. In addition, the tilt angle is a constant value, which can be easily obtained through experiments. Therefore, when we obtain the initial position of the coil in the tilt state, we can directly calculate the tilt error through Equation (6).

#### 2.2.3. Comprehensive Eccentricity Model

Based on the above analysis, the *δ_e_* caused by the coil center offset and the *δ_β_* caused by coil inclination present a periodic change with the rotation angle *θ*; the comprehensive eccentricity *δ* can be expressed as a related function with the rotation angle *θ*. Due to a tiny tilt angle *β*, the *δ* is approximately equal to the sum of the radial and tilt eccentricity. Therefore:

(7) $$\delta \approx \delta_{e}+\delta_{\beta }$$

It can be seen from Equations (3), (6), and (7) that the comprehensive eccentricity is related to the radial parameter and the tilt parameter. In fact, since the tilt angle always exists, the radial eccentricity is not directly obtained. Similar to the analysis in Section 2.2.1, the comprehensive eccentricity can be directly obtained from the initial position of the coil in the inclined state, which is expressed as Equation (8):

(8) $$\left\{\begin{array}{l} \delta_{X}=e^{\prime }\left[\cos(\alpha^{\prime \prime }+\theta )-\cos(\alpha^{\prime \prime })\right]\\ \delta_{Y}=e^{\prime }\left[\sin(\alpha^{\prime \prime }+\theta )-\sin(\alpha^{\prime \prime })\right] \end{array}\right.$$

where the comprehensive model parameters are *e*′ and *α*″. Consistent with the previous derivation, it can be directly calculated by the initial position of the coil. Therefore, we can obtain the *δ* at the beginning. If the tilt angle is determined, the tilt eccentricity *δ_β_* can be obtained by Equation (6). Finally, according to Equation (7), the radial eccentricity *δ_e_* can be approximately calculated without considering the tilt factor.

After analyzing the source of eccentricity in the wire-traction system, the eccentric model was established. It can intuitively give the main factors affecting the eccentricity. After determining the main parameters in the model, the eccentricity can be calculated directly. If the model has an accurate prediction on the eccentricity, the eccentric law of the whole rotation can be obtained according to the initial position of the coil during the actual correction, and, then, the correction can be implemented.

### 2.3. Parameter Calibration by Image Processing

#### 2.3.1. Central Coordinate Extraction

In this paper, a prediction method for measuring coil eccentricity by using eccentricity models is proposed, which requires calibration of the parameters, including coil center, rotational center, and tilt angle. To address this, an algorithm for coil center detection was designed based on micro image processing technology. The processes of this algorithm are as follows:

Step 1: Acquisition, graying, and filtering of microscopic images. Firstly, Figure 6a shows the collection of the microscopic coil image using the vertical camera; then, the color microscopic image is grayed by,

(9) Gray(x,y)=αR(x,y)+βG(x,y)+γB(x,y)

where *R*(*x*, *y*)*, G*(*x*, *y*)*,* and *B*(*x*, *y*) are three components of *RGB*, and *α* = 0.299, *β* = 0.578, *γ* = 0.114 are coefficients. Proper image filtering can enhance image quality. Since the median filtering can effectively protect the edges and reduce the degree of blur while suppressing noise, this paper uses the median filtering to preprocess the microscopic image by Equation (10), and Figure 6b shows the filtering result of the coil microscopic image by

(10) $$g(x,y)=\underset{i,j\in FT}{MF}(f(x+i,y+j))$$

where *MF* is the median function, and *FT* is the filter template.

Step 2: Segmentation processing of microscopic image. An adaptive threshold segmentation algorithm (Otsu), which is a maximum inter-class variance method [21], is used for image segmentation. In Otsu, the inter-class variance is a function of the threshold value. The threshold value that makes the variance reach the maximum is the segmentation threshold value *T*. It can adaptively determine the threshold value, maximize the variance value between the target and the background area, and achieve a good segmentation effect. The image is binarized based on Otsu by

(11) $$f(x)=\left\{\begin{array}{c} 0\\ 255 \end{array}\right.\begin{array}{c} x\leq T\\ x>T \end{array}$$

where 0 represents the grayscale of black pixels, and 255 represents the grayscale of white pixels. Figure 6c shows the result after threshold segmentation of the coil micrograph.

Step 3: Morphological processing of microscopic images. In the binary image after threshold segmentation, there may be tiny holes at the white boundary of the target area. In order to fill these holes, the microscopic image is subjected to morphological closed operation, which can also significantly improve the smoothness of the boundary. Figure 6d shows the processing result of the coil microscopic image.

Step 4: Extract the maximal connected area in the microscopic image. After the threshold segmentation, there are still large black interference areas in the white target area of the binary image. In order to eliminate these interference areas, the gray values of all pixels in the microscopic image are inverted, then the maximum connected area in the image is extracted, and the gray values of all pixel points outside this connected area are set to 0 so that the interference areas in the target can be eliminated. At this time, the entire black connected area becomes the target area; Figure 6e shows the processing result of coil microscopic image.

Step 5: Edge detection of the target area in the microscopic image. In order to obtain a series of important edge points and facilitate the subsequent use of the circle feature extraction algorithm to fit the circle and extract the image coordinates of the circle center, it is necessary to perform edge detection on the target area obtained in Step 4. In this paper, the Canny operator, which is superior in noise suppression and edge positioning accuracy, was selected for edge detection of microscopic images. Figure 6f shows the result of edge detection of coil microscopic images.

Step 6: Extract the circle center based on the circle feature extraction algorithm. The accuracy of circle feature extraction has a great impact on the calculation result of eccentricity error. Compared with other algorithms, the least square fitting method can obtain the circle parameters by only circulating the edge points once, with low time complexity, fast calculation speed, and high accuracy and robustness. Therefore, this paper chose the least square fitting algorithm to extract the circle feature and calculate the image coordinates of the coil center. The algorithm principle of the least-square method to calculate the center is as follows:

The equation of circle is defined as

(12) $${\left(x-a\right)}^{2}+{\left(y-b\right)}^{2}=r^{2}$$

where (*a*, *b*) represents its center, and *r* represents its radius.

The residual error is defined as

(13) $$\delta_{i}={\left(x_{i}-a\right)}^{2}+{\left(y_{i}-b\right)}^{2}-r^{2}$$

where (*x_i_*, *y_i_*) represents coordinates of edge points obtained by edge detection, and *i* represents the index of edge points.

The sum of residual error square of circle fitting is defined as

(14) $$Q=\sum_{i=1}^{num}\delta_{i}^{2}=\sum_{i=1}^{num}{\left[{\left(x_{i}-a\right)}^{2}+{\left(y_{i}-b\right)}^{2}-r^{2}\right]}^{2}$$

where *num* represents the number of edge points.

According to the principle of least-square method, *Q* calculates the partial derivative of *a*, *b*, and *r*, respectively, and the partial derivative is equal to 0.

(15) $$\left\{\begin{array}{l} \frac{\partial Q}{\partial a}=-4\sum_{i=1}^{num}\left[{\left(x_{i}-a\right)}^{2}+{\left(y_{i}-b\right)}^{2}-r^{2}\right](x_{i}-a)=0\\ \frac{\partial Q}{\partial b}=-4\sum_{i=1}^{num}\left[{\left(x_{i}-a\right)}^{2}+{\left(y_{i}-b\right)}^{2}-r^{2}\right](y_{i}-b)=0\\ \frac{\partial Q}{\partial r}=-4\sum_{i=1}^{num}\left[{\left(x_{i}-a\right)}^{2}+{\left(y_{i}-b\right)}^{2}-r^{2}\right]r=0 \end{array}\right.$$

We get:

(16) $$\left\{\begin{array}{l} a^{2}-2\bar{x}a+b^{2}-2\bar{y}b-r^{2}+\bar{x^{2}}+\bar{y^{2}}=0\\ \bar{x}a^{2}-2\bar{x^{2}}a+\bar{x}b^{2}-2\bar{xy}b-\bar{x}r^{2}+\bar{x^{3}}+\bar{xy^{2}}=0\\ \bar{y}a^{2}-2\bar{xy}a+\bar{y}b^{2}-2\bar{y^{2}}b-\bar{y}r^{2}+\bar{x^{2}y}+\bar{y^{3}}=0 \end{array}\right.$$

where $\bar{x^{m}y^{n}}=\frac{1}{num}\sum_{i=1}^{num}x_{i}^{m}y_{i}^{n}$ substitutes the above formula and obtains a binary linear equation:

(17) $$\left\{\begin{array}{c} ({\bar{x}}^{2}-\bar{x^{2}})a+(\bar{x}\bar{y}-\bar{xy})b=(\bar{x^{2}}\bar{x}+\bar{x}\bar{y^{2}}-\bar{x^{3}}-\bar{xy^{2}})/2\\ (\bar{x}\bar{y}-\bar{xy})a+({\bar{y}}^{2}-\bar{y^{2}})b=(\bar{x^{2}}\bar{y}+\bar{y}\bar{y^{2}}-\bar{x^{2}y}-\bar{y^{3}})/2 \end{array}\right.$$

The parameters *a*, *b*, and *r* of the fitting circle equation are then calculated:


(18) $$\left\{\begin{array}{l} a=\frac{\left(\bar{x^{2}}\bar{x}+\bar{x}\bar{y^{2}}-\bar{x^{3}}-\bar{xy^{2}})({\bar{y}}^{2}-\bar{y^{2}})-(\bar{x^{2}}\bar{y}+\bar{y}\bar{y^{2}}-\bar{x^{2}y}-\bar{y^{3}})(\bar{x}\bar{y}-\bar{xy}\right)}{2({\bar{x}}^{2}-\bar{x^{2}})({\bar{y}}^{2}-\bar{y^{2}})-2{\left(\bar{x}\bar{y}-\bar{xy}\right)}^{2}}\\ b=\frac{\left(\bar{x^{2}}\bar{y}+\bar{y}\bar{y^{2}}-\bar{x^{2}y}-\bar{y^{3}})({\bar{x}}^{2}-\bar{x^{2}})-(\bar{x^{2}}\bar{x}+\bar{x}\bar{y^{2}}-\bar{x^{3}}-\bar{xy^{2}})(\bar{x}\bar{y}-\bar{xy}\right)}{2({\bar{x}}^{2}-\bar{x^{2}})({\bar{y}}^{2}-\bar{y^{2}})-2{\left(\bar{x}\bar{y}-\bar{xy}\right)}^{2}}\\ r=\sqrt{a^{2}-2\bar{x}a+b^{2}-2\bar{y}b+\bar{x^{2}}+\bar{y^{2}}} \end{array}\right.$$

Figure 6g,h respectively show the results of edge contour fitting and circle feature extraction of the coil microscopic image. Among them, the partial area shows that the extracted circle had a high degree of fit with the actual outer circle of the coil.

The above is the extraction algorithm flow of coil circle center. If the microscope image sequence of the eccentric coil is input, the center coordinates of the image all can be extracted, as shown in Figure 6i. According to this method, the coil center can be obtained. When there are many images in the sequence, the points of the coil center can be fitted into a circle, and the center of this circle is the actual rotation center of the coil. By this approach, the actual rotation center can be calculated. In addition, due to the calculation principle of the least-square method, the pixel of the theoretical center fitted is not generally integral in the subsequent experiments.

#### 2.3.2. Angle Measurement

In order to measure the tilt angle, an algorithm for angle detection was designed based on image processing technology in this paper. Figure 7a is a horizontal coil image during rotation, and Figure 7b is the result of the edge detection. The image processing from Figure 7a,b is consistent with step 1 to step 5 in the center extraction algorithm. Figure 7c shows the result of straight-line detection for the edge of the microscopic image. In this paper, the Hough transform was used to detect the straight-line region in the microscopic image [22]. The two sides of the outer circle of the coil and the two sides of the central column of the coil were detected. Four boundary straight lines were extracted from each image. The slope and tilt angle of the straight lines were calculated according to the equation, and the average value was taken as the tilt angle of the coil in the image. When the coil rotates one turn in the tilted state, an image sequence is acquired and the tilt angles are calculated. The maximum among them was taken as the tilt angle of the coil.

### 2.4. A Coarse-Precise Correction

According to the establishment of eccentricity model, the comprehensive eccentricity includes radial and tilt eccentricity. Both are systematic biases, which could be decreased by reasonable correction methods. The radial one accounts for a large part, but the tilt one is tiny. They can be compensated separately in order to ensure that the eccentricity value fluctuates slightly near the starting position. Thus, a coarse–precise correction for coil eccentricity was designed in this paper. This method relies on eccentric compensation models and the five-axis motion control system to correct eccentricity, which realizes a correction method based on the hardware and algorithm. The structure of this system is shown in Figure 1, and its correction flow are mainly as follows:

Step 1: Calibration of model parameters. Before the correction, the actual rotation center (*u*,*v*) and the tilt angle *β* are detected from the eccentric images using proposed algorithms, and the initial center coordinates (*x*_0_,*y*_0_) can be obtained by detecting the initial coil. After determining parameters, they are put into the error models to obtain comprehensive and tilt eccentricity.

Step 2: Centering correction. According to the comprehensive model, the difference between the actual rotation center of the rotary stage and the actual center of coil base can be calculated. The two-DOF manual precise motion stages B1 and B2 driven by the micrometer can be used to center the coil. The center line of the coil can be adjusted to coincide with the rotation axis of the rotary stage. At this time, the eccentricity of the coil is greatly decreased. In addition, the centering compensation model can be express as

(19) $$\left\{\begin{array}{l} CM_{B1}=\lambda \sqrt{{\left(y_{0}-v\right)}^{2}+{\left(x_{0}-u\right)}^{2}}\cos(\arctan((y_{0}-v)/(x_{0}-u)))\\ CM_{B2}=\lambda \sqrt{{\left(y_{0}-v\right)}^{2}+{\left(x_{0}-u\right)}^{2}}\sin(\arctan((y_{0}-v)/(x_{0}-u))) \end{array}\right.$$

where *CM* represents the compensation models. *λ* = 2.3 μm/pixel is the conversion of the object and image. It can be obtained by calibrating the displacement difference of the chessboard corners fixed on the stage in the image after controlling the electronic motion stage to move a fixed distance.

Step 3: Precision correction. After the centering correction, the radial eccentricity is almost tiny, but the tilt eccentricity and residual error still affect the normal operation of the wire-traction. In the previous analysis of the system workflow, the ideal wire-traction environment is where the coil is fixed at the initial position and rotates without eccentricity after moving to the area to be measured. Therefore, the precision correction uses the accurate electronic control motion stages A1 and A2 to control the coil center to move to the initial position of the coil in real time in order to decrease the eccentricity caused by the coil tilt. The entire process of precision correction is completed until the end of wire-traction. According to the tilt model, the precision compensation models of the electronic motion stages are established by

(20) $$\left\{\begin{array}{l} CM_{A1}=\lambda \delta_{\beta X}\\ CM_{A2}=\lambda \delta_{\beta Y} \end{array}\right.$$

## 3. Results

In this section, the calibration of model parameters, the accuracy of the eccentricity models, and the effect of the coil eccentricity correction are carried out through experiments. The wire-traction system in Figure 1 was used for the experiments. In the system, the main technical parameters of the five-axis motion system are described as the combination of the two-directional manual stage (B1 andB2) LY50-LM, the two-directional motorized stage (A1 and A2), A032, and the motorized rotary stage (A3) HWDD-WZ-65-112. The accuracy of the manual stage in micrometers was 0.01 mm. The resolution of the motorized motion stage was 0.000625 mm, the repetitive positioning accuracy was 0.002 mm, and the maximum operating speed was 25 mm/s. The resolution of the rotary stage was 0.001°, and the repetitive positioning accuracy was ±0.04°. The resolution of the cameras used in the microvision system is 2448 × 2048 pixel, and the optical magnification is 1.5×. The microvision system composed of two CMOS cameras was used to capture the microscope images of the coil from the top and side views.

### 3.1. Calibration Results

In order to accurately calibrate the model parameters, ten coils with a uniform shape and exceptional surface processing quality were selected as experimental samples. In the experiment, the coils were all rotated for one cycle, and their microscope images were collected every 5° from the initial position. Table 1 shows parts of the vertical and horizontal microscope images collected during rotation. The actual rotation angle of the rotary stage was set as *θ.* It can be seen that the coil had significant eccentricity and inclination. We used the image processing flow introduced in Figure 6 to extract the center coordinates of the eccentric coil and calculate its actual rotary center. At the same time, the tilt angle of the eccentric coil was measured based on the angle detection introduced in Figure 7. The model parameters were determined as shown in Table 2. The result shows that the actual rotation center and tilt angle of the coil were approximately constant, because the rotation center of this system was moved to the invariant area before each wire-traction, and the coil was fixed by using the same base. This is consistent with the results of our previous analysis. Finally, the actual rotation center of the coil was (1145,1267) in the pixel coordinate system, and the mean tilt angle of the coil was 0.19°.

### 3.2. Model Accuracy and Eccentricity Correction

To validate the measurement accuracy of the eccentricity models and assess the effectiveness of the proposed correction method in this paper, the coarse–precise correction approach was conducted to compensate eccentric coils on the basis of calibrating model parameters. In the experiment, the initial center coordinates of the above-mentioned coils were extracted to input eccentricity models in order to predict the comprehensive and tilt eccentricity. Additionally, their microscope images were collected every 5°. Image processing, illustrated in Figure 6, was used to obtain the central coordinates of the eccentric coil as a comparison with the prediction of the models to verify the measurement accuracy of the models.

Figure 8a,b respectively show the comparison between the results calculated by the comprehensive eccentricity model and the eccentric curve obtained by the visual detection in the *X* and *Y* directions. Similarly, Figure 8c,d also show the comparison of tilt eccentricity model after centering correction. It can be seen that the models demonstrated a fine prediction of the eccentricity.

The root mean square error (RMSE) has the advantage of directly reflecting the residual error between the model and the actual experiment. Thus, it can be used to evaluate the prediction accuracy of error models. [23] Table 3 shows the RMSE of the residual error in Figure 8. From the table, the lowest RSME of the comprehensive model was 0.3143, and the other model was 0.7301. The deviation of converting them into object space was 0.8995 μm and 1.6792 μm, respectively. The accuracy of the proposed models in measuring eccentricity is evident. This suggests that eccentricity models have the potential to replace the entire visual detection process.

In addition, Figure 8a,b show that the maximal bias was about 100–140 pixels. After the centering correction, shown in Figure 8c,d, the eccentricity was greatly decreased to around 20 pixels. According to the previous analysis, the residual error was almost tilt eccentricity, which could be compensated by following precision correction. In addition, the image coordinates of coil center were extracted every 5° as the final bias of the coil. Figure 9a,b indicate that the coil center after precision correction was distributed in a scattered manner near the coil initial position after centering correction, and the overall eccentricity did not exceed three pixels.

More specifically, the final residual error in the *X* direction was generally in the interval from −0.77 pixels to 2.08 pixels, and it was in the interval from −0.37 pixels to 2.53 pixels in the *Y* direction. We can observe that the actual bias in the *X* direction was in the interval from −1.77 μm to 4.78 μm, and it was in the interval from −0.85 μm to 5.82 μm in the *Y* direction. Finally, the coil had a tiny position fluctuation near the initial position, and the maximal eccentricity was less than 6 μm. Figure 9c,d show the eccentricity curve of one coil in the *X* and *Y* directions before and after correction. Obviously, it can be intuitively seen that the eccentricity was greatly decreased. The average compensation amplitude of ten coils was calculated to be about 99.6%, which verifies the effectiveness of this correction method.

### 3.3. Discussion

Eccentricity measurement is an important and fundamental issue in the field of mechanical engineering [24]. The motivation of this paper was to measure and correct the rotational eccentricity of the coil during automatic traction to enhance the micromanipulation accuracy and achieve an optimal automatic wire-traction effect.

In terms of eccentricity measurement, this paper presents a novel method for measuring eccentricity using eccentricity models and microvision. With advancements in computer technology and algorithm optimization, vision-based measurement techniques have become highly accurate and efficient while also offering the advantage of being non-contact. Moreover, microscopic vision can process images at the pixel or sub-pixel level, thus providing high resolution and precision [24]. The proposed method employed a five-megapixel camera that achieves an object image conversion rate of 2.3 μm/pixel, which was considered to be the measurement accuracy due to the high-performance microscopic image algorithm. In addition, eccentricity models are developed based on visual measurement and are further calibrated to improve measurement speed. By adopting a single-point measurement mode, the proposed method reduced measurement complexity. Furthermore, the method was embedded in the system and was well-suited for measuring micron-level objects.

In terms of eccentricity correction, the error sources of coil rotation eccentricity were analyzed first. It was found that radial bias was the major contributor to rotation eccentricity. Therefore, a centering correction method was employed to compensate for the radial bias, thus resulting in a significant reduction in eccentricity. The residual error, mainly caused by tilt error, was compensated by precision correction, which effectively reduced the residual bias to within 6 μm. The compensation amplitude of this method reached 99.6%, which significantly enhances the manipulating accuracy of the system. This correction method is both reasonable and time-saving, and it can be integrated into the wire-traction flow for ease of use. Overall, the proposed correction method offers a real-time and effective solution to reduce the impact of coil rotation eccentricity on the manipulating accuracy of the system.

However, although the proposed correction method effectively reduced the eccentricity to within 6 μm, it is important to note that residual errors still exist. In future work, it is necessary to consider other sources of errors, such as errors in visual detection caused by the nonorthogonality of the camera’s optical axis with the object plane [25], and errors in correcting hardware systems caused by the nonorthogonality of the multiaxis [26]. Moreover, the accuracy of the feature extraction algorithm, which is used to extract features of coil images to determine related parameters, will also directly impact the measurement results. It is, therefore, necessary to conduct experimental analysis to verify the accuracy of the detection algorithm in next work. Additionally, the effect on the wire-traction after correction is not presented in this paper. To further verify the effectiveness of the system, some traction renderings are expected to be included in future work.

## 4. Conclusions

In this paper, a novel measurement for the coil eccentricity based on eccentricity models and microvision, as well as correction combining hardware and an algorithm, have been proposed in order to compensate for rotational eccentricity in the wire-traction micromanipulation system. This work is summarized as follows:

1. The radial and tilt eccentricity models were established from the error sources of the coil rotational eccentricity.
2. The measurement method by models and microvision had an accurate prediction on the coil eccentricity.
3. The residual error of the coil was reduced within 6 μm using a coarse–precise correction approach, thereby achieving a compensation amplitude of about 99.6%. The proposed method can effectively decrease coil eccentricity and enhance the control accuracy of the wire-traction system.

## Figures and Tables

**Figure 1:**
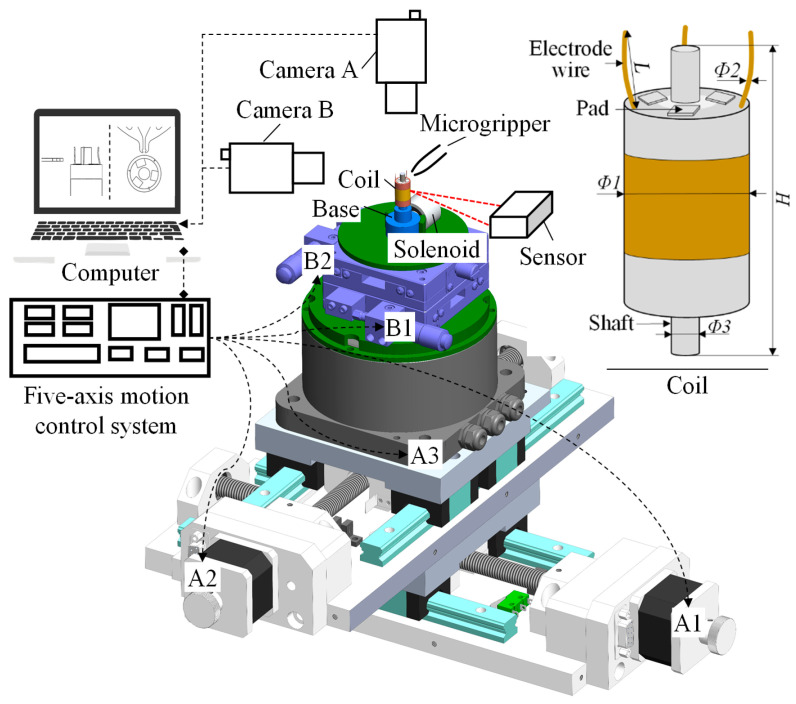
Automatic wire-traction system and the coil’s structure model. A1: *X*-axis electronic motion stage; A2: *Y*-axis electronic motion stage; A3: *R*-axis rotary stage; B1: *X*-axis manual motion stage; B2: *Y*-axis manual motion stage.

**Figure 2:**
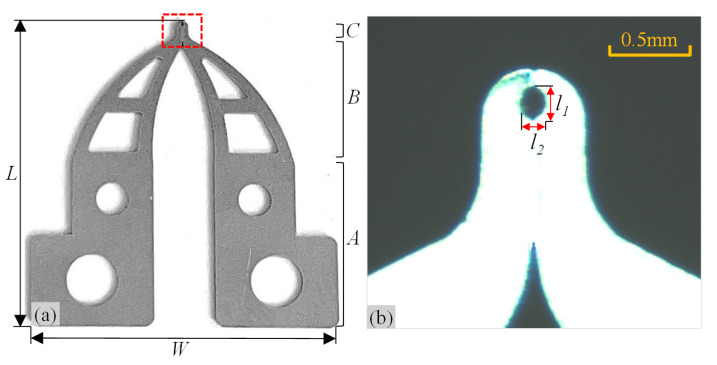
Structure and dimensions of the microgripper. (**a**) Symmetrical microgripper. A: Assembly area; B: Support area; C: Clamping area; (**b**) Enlarged area of red dotted line frame.

**Figure 3:**
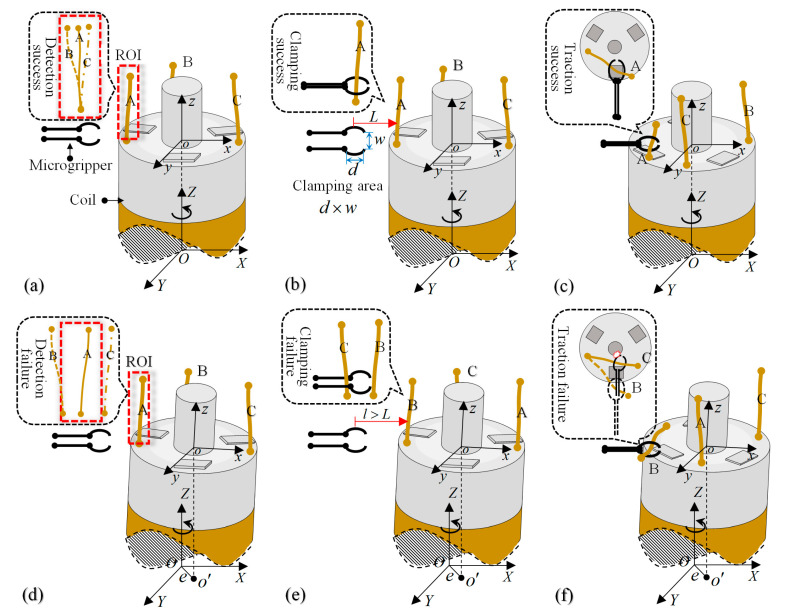
Analyzing the influence of eccentricity on wire-traction system. (**a**) Successful wire detection under ideal conditions. *OXYZ* is the rotational coordinate system. *oxyz* is the coordinate system of the coil; (**b**) Successful clamping for the wire under ideal conditions. *L* represents the distance between the center of the tip of the microgripper and the wire in theory. Clamping area *d* × *w* is 0.2 mm × 0.5 mm; (**c**) Successful wire-traction under ideal conditions; (**d**) Detection failure under eccentric condition. *o*′ is the projection of coil center and *e* is the eccentricity; (**e**) Clamping failure under eccentric condition: *l* is the actual distance between the center of the tip of the microgripper and the wire under eccentric condition; (**f**) Traction failure under eccentric condition.

**Figure 4:**
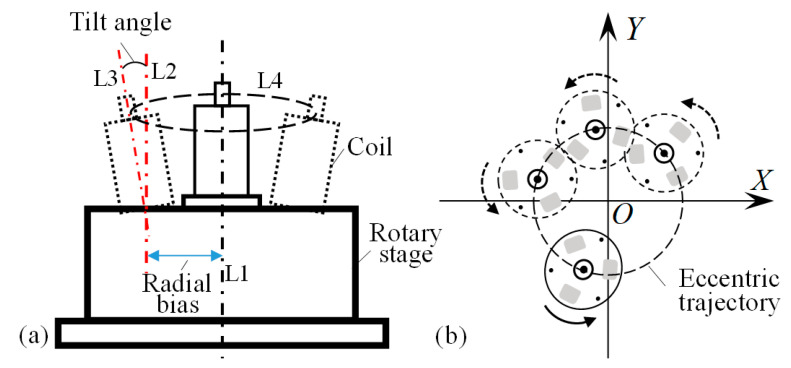
Eccentricity of coil in wire-traction system. (**a**) Analysis of eccentricity sources: L1 is the axis of rotation, L2 is the eccentric axis of coil, L3 is the tilt axis of coil, and L4 is the eccentric trajectory; (**b**) Eccentric trajectory of coil.

**Figure 5:**
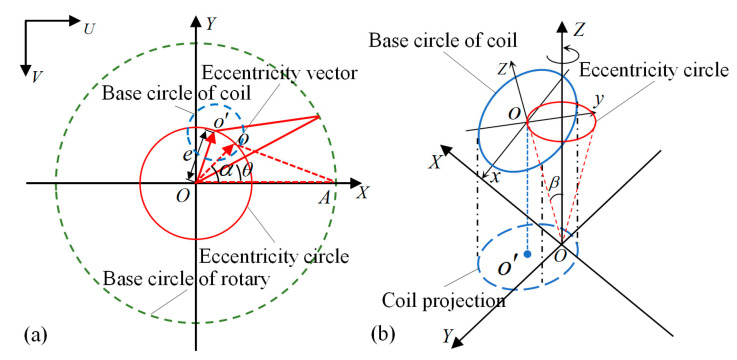
The coil’s eccentricity model. (**a**) Radial eccentricity model in pixel coordinate system; (**b**) Tilt eccentricity model in spatial coordinate system.

**Figure 6:**
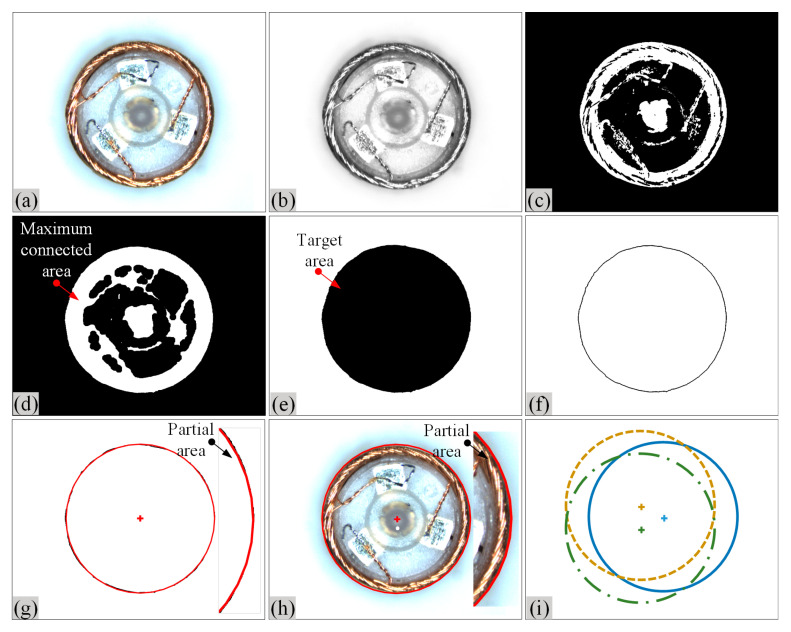
Algorithm flow of coil center detection. (**a**) Coil microscopic image; (**b**) Gray-Level image after median filtering; (**c**) Binary image after threshold segmentation; (**d**) Coil microscopic image after morphological processing; (**e**) Target area image after preprocessing; (**f**) Edge detection result; (**g**) Edge contour fitting result (red cross is the center of the fitting circle); (**h**) Circle feature extraction result of coil microscopic image (red cross is the center of the fitting circle); (**i**) Circle extraction results of microscopic image sequence (blue solid line, yellow dotted line, and green dotted line represent circle extraction results of image sequence with coil rotation of 0°, 120° and 240°, respectively).

**Figure 7:**
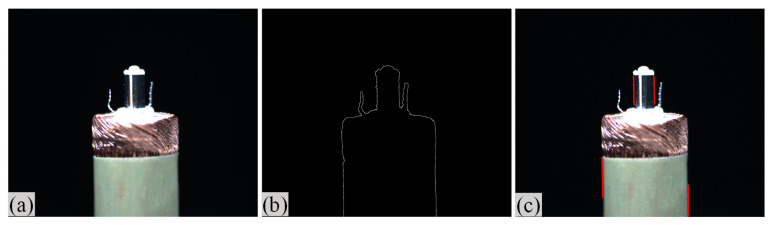
Algorithm flow of coil angle detection. (**a**) Coil microscopic image; (**b**) Edge detection; (**c**) Line detection (Red lines are the result of straight-line extraction).

**Figure 8:**
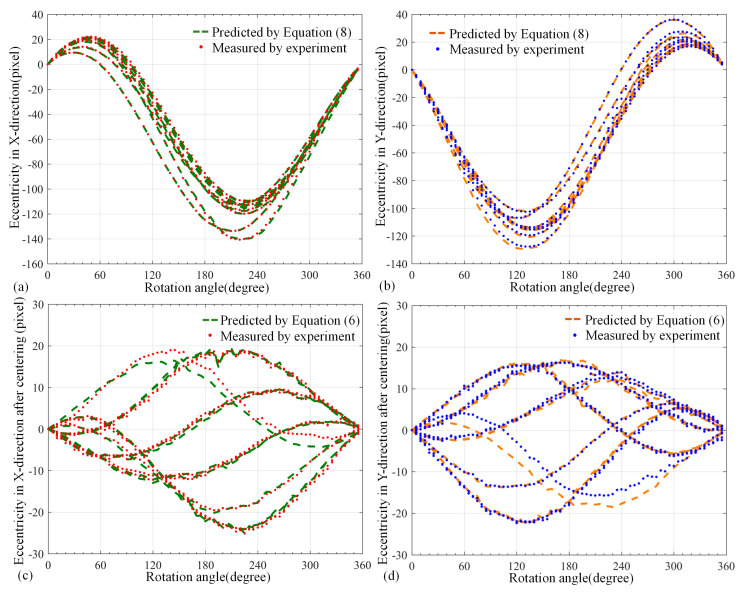
The curve of coil eccentricity. (**a**) Comprehensive eccentricity in *X* direction; (**b**) Comprehensive eccentricity in *Y* direction; (**c**) Tilt eccentricity in *X* direction after centering correction; (**d**) Tilt eccentricity in *Y* direction after centering correction.

**Figure 9:**
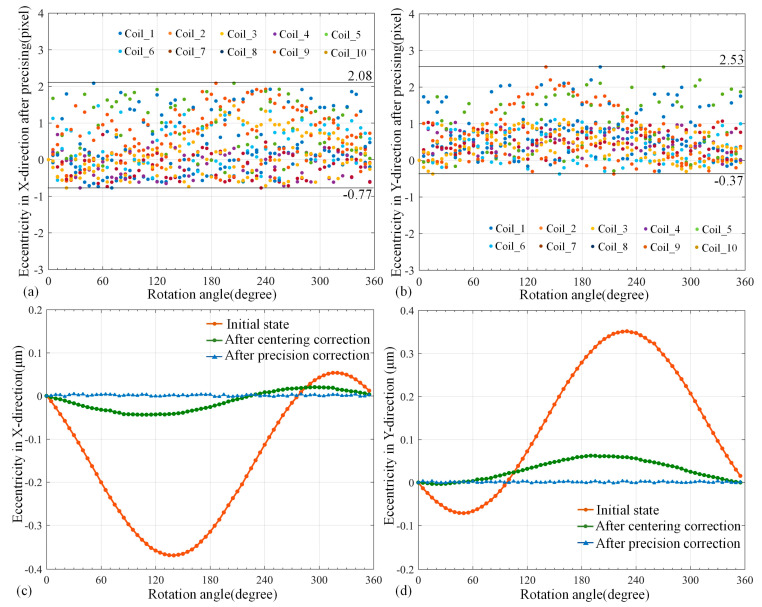
The curve of coil eccentricity. (**a**) Eccentricity in *X* direction after precision correction; (**b**) Eccentricity in *Y* direction after precision correction; (**c**) Comparison of eccentricity error in *X* direction under three states of any coil; (**d**) Comparison of eccentricity in *Y* direction under three states of any coil.

**Table 1:** Partial microscopic images of the coil during rotation.

**Vertical**	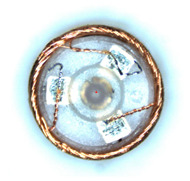	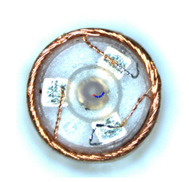	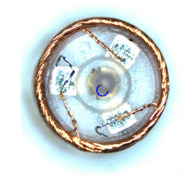	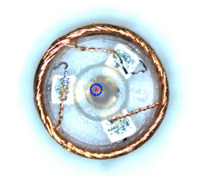
**Angle**	*θ* = 0°	*θ* = 120°	*θ* = 240°	*θ* = 360°
**Horizontal**	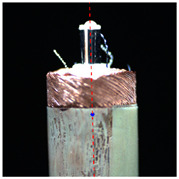	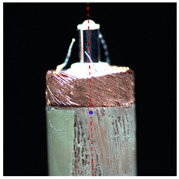	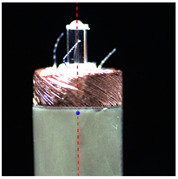	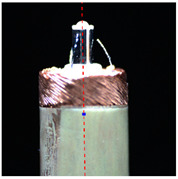
**Angle**	*θ* = 0°	*θ* = 90°	*θ* = 180°	*θ* = 270°

*θ* represents rotation angle of the coil.

**Table 2:** Calibration of model parameters.

Coils	Coil_1	Coil_2	Coil_3	Coil_4	Coil_5
**Rotation center (pixel)**	(1144,1267)	(1144,1268)	(1145,1271)	(1144,1267)	(1145,1267)
**Tilt angle (°)**	0.21	0.18	0.19	0.20	0.21
**Coils**	Coil_6	Coil_7	Coil_8	Coil_9	Coil_10
**Rotation center (pixel)**	(1144,1266)	(1145,1268)	(1145,1266)	(1145,1267)	(1145,1267)
**Tilt angle (°)**	0.17	0.20	0.20	0.20	0.17

**Table 3:** Eccentricity model accuracy by RMSE.

Models	RSME (*X*-Direction)	RSME (*Y*-Direction)
Comprehensive model	0.3911	0.3143
Tilt model	0.7301	0.7480

## Data Availability

The data presented in this study are available on request from the corresponding author. The data are not publicly available due to the personal privacy of participating researchers.

## References

[1] Guo S.X., Sugimoto K., Hata S. A human scale tele-operating system for microoperation. Proceedings of the 2001 IEEE International Symposium on Computational Intelligence in Robotics and Automation.

[2] Wason J.D., Wen J.T., Gorman J.J., Dagalakis N.G. (2012). Automated multiprobe microassembly using vision feedback. IEEE Trans. Robot..

[3] Sano T., Yamamoto H. Study of micromanipulation using stereoscopic microscope. Proceedings of the 17th IEEE Instrumentation and Measurement Technology Conference.

[4] Fabian R., Tyson C., Tuma P.L., Pegg I., Sarkar A. (2018). A horizontal magnetic tweezers and its use for studying single DNA molecules. Micromachines.

[5] Richa R., Balicki M., Sznitman R., Meisner E., Taylor R., Hager G. (2012). Vision-based proximity detection in retinal surgery. IEEE Trans. Biomed. Eng..

[6] Roland P., Miguel A.N., Perez J.A., Javier C. (2011). Geared PM coreless motor modelling for driver’s force feedback in steer-by-wire systems. Mechatronics.

[7] Xu X., Zhou K.K., Zou N.N., Jiang H. (2015). Hierarchical control of ride height system for electronically controlled air suspension based on variable structure and fuzzy control theory. Chin. J. Mech. Eng..

[8] Wang Y.Z., Ma G.D., Zhang C.C. (2015). The wire traction microgripper design and experiments for stereo light microscope. Int. J. Appl. Electromagn. Mech..

[9] Wang Y., Long C., Sun Y. System design based on microscopic vision with stereo light microscope for gripping microscopic objects. Proceedings of the 2017 IEEE International Conference on Mechatronics and Automation (ICMA).

[10] Wang Y., Chen J., Qu D. (2022). Design, analysis and experimental investigations of a double-arm based micro-gripper for thin and flexible metal wires manipulation. Micromachines.

[11] Geckeler R.D., Link A., Krause M., Elster C. (2014). Capabilities and limitations of the self-calibration of angle encoders. Meas. Sci. Technol..

[12] Li Y.-T., Fan K.-C. (2017). A novel method of angular positioning error analysis of rotary stages based on the abbe principle. Proc. Inst. Mech. Eng. Part B J. Eng. Manuf..

[13] Yu Y., Dai L., Chen M.-S., Kong L.-B., Wang C.-Q., Xue Z.-P. (2020). Calibration, compensation and accuracy analysis of circular grating used in single gimbal control moment gyroscope. Sensors.

[14] Du Y., Yuan F., Jiang Z., Li K., Yang S., Zhang Q., Zhang Y., Zhao H., Li Z., Wang S. (2021). Strategy to decrease the angle measurement error introduced by the use of circular grating in dynamic torque calibration. Sensors.

[15] Jia H.-K., Yu L.-D., Zhao H.-N., Jiang Y.-Z. (2019). A new method of angle measurement error analysis of rotary encoders. Appl. Sci..

[16] Chen S.-P., Wang Z.-Z., Yu H., Lin L.-Q. (2018). Research on automatic compensation technology for eccentricity of grinding wheel. Int. J. Precis. Eng. Manuf..

[17] Lou Z.-L., Xue P.-F., Zheng Y.-S., Fan K.-C. (2018). An analysis of angular indexing error of a gear measuring machine. Appl. Sci..

[18] Lou Z.-F., Hao X.-P., Cai Y.-D., Lu T.-F., Wang X.-D., Fan K.-C. (2019). An embedded sensor system for real-time detecting 5-DOF error motions of rotary stages. Sensors.

[19] Lou Z.-F., Liu L., Zhang J.-Y., Fan K.-C., Wang X.-D. (2021). A self-calibration method for rotary tables’ five degrees-of-freedom error motions. Measurement.

[20] Huang X., Wang Z., Shen B., Lei P. (2021). Research on self-aligning flanges based on piezoelectric actuators applied to precision grinding machines. Micromachines.

[21] Goh T.Y., Basah S.N., Yazid H., Aziz Safar M.J., Ahmad Saad F.S. (2018). Performance analysis of image thresholding: Otsu technique. Measurement.

[22] Chao Y., Zhao G., Huang S. A novel parallel beeline detection algorithm based on improved Hough transform. Proceedings of the Eighth International Symposium on Multispectral Image Processing and Pattern Recognition (MIPPR)-Automatic Target Recognition and Navigation.

[23] Lee I.-H., Mahmood M.-T., Choi T.-S. (2015). Robust focus measure operator using adaptive Log-Polar mapping for three-dimensional shape recovery. Microsc. Microanal..

[24] Fang Y., Wang X., Xin Y., Luo Y. (2022). Sub-pixel dimensional and vision measurement method of eccentricity for annular parts. Appl. Opt..

[25] Huang F.Y., Shen X.J., Wang Q., Wang Z.B., Hu W.G., Shen H.B., Li L. (2013). Correction method for fisheye image based on the virtual small-field camera. Opt. Lett..

[26] Wang Y., Liu J., Chen H., Chen J., Lu Y. (2021). Orthogonality measurement of three-axis motion trajectories for micromanipulation robot systems. Micromachines.

